# Evaluation of Antiradical and Antioxidant Activities of Lipopeptides Produced by *Bacillus subtilis* Strains

**DOI:** 10.3389/fmicb.2022.914713

**Published:** 2022-06-20

**Authors:** Elodie Dussert, Mélissa Tourret, Chloé Dupuis, Alexandre Noblecourt, Josette Behra-Miellet, Christophe Flahaut, Rozenn Ravallec, François Coutte

**Affiliations:** ^1^Univ. Lille, Univ. Artois, UMRT 1158 BioEcoAgro - Bénéfice santé d'hydrolysats de protéines et coproduits agro-alimentaires, Institut Charles Viollette, Lille, France; ^2^Univ. Lille, UMRT 1158 BioEcoAgro - Métabolites secondaires d'origine microbienne, Institut Charles Viollette, Lille, France; ^3^LIPOFABRIK, Lesquin, France

**Keywords:** antioxidant, antiradical, *Bacillus subtilis*, lipopeptides, reactive oxygen species

## Abstract

This study investigated the antiradical and antioxidant potential of the three families of lipopeptides (i.e., surfactin, mycosubtilin, and plipastatin/fengycin) produced by *Bacillus subtilis* strains. The antiradical/antioxidant activities of highly purified lipopeptides were studied in acellular models using a 2,2-diphenyl-1-picrylhydrazyl (DPPH) radical, superoxide anion (${\text{O}}_{2}^{.-}$), hydrogen peroxide, (H_2_O_2_) and hydroxyl radical (HO^.^). At a lipopeptide concentration of 500 mg.L^−1^, the maximum inhibition of DPPH reached 22.88% (obtained for plipastatin). Moreover, the scavenging effects of ${\text{O}}_{2}^{.-}$, H_2_O_2_, and HO^.^ at the highest concentration tested (250 mg.L^−1^) were found to be 6, 21, and 3% for surfactin, 19, 9, and 15% for mycosubtilin, 21, 18, and 59% for plipastatin, 21, 31, and 61% for the mixture of surfactin/plipastatin, and 13, 16, and 15% for the mixture of surfactin/mycosubtilin, respectively. These results showed that plipastatin was the best candidate due to its antioxidant activities.

## Introduction

*Bacillus subtilis* is a very well-studied bacterial species used in industry in many sectors because of its ability to produce many molecules of interest. It is known to have excellent protein secretion ability, making it an important host for the production of some molecules such as proteins, vitamins, and antibiotics. In pharmaceuticals, menaquinone-7, a vitamin produced by *B. subtilis*, has shown beneficial effects in osteoporosis (Knapen et al., 2013) and can reduce the risk of coronary heart disease (Geleijnse et al., 2004). In the food industry, proteases from *B. subtilis* can be used in the production of cheese (milk-clotting) (Meng et al., 2018) and as a meat tenderizer (Bureros et al., 2020). In the poultry industry, *B. subtilis*-fermented products have potential for development as feed additives and use as possible substitutes for antibiotics to treat infection by *Clostridium perfringens* (Cheng et al., 2018). In the phytosanitary sector, *B. subtilis* has been used for years as a biopesticide to control plant pathogenic fungi; this property is directly related to the production of antifungal lipopeptides (Ongena and Jacques, 2008).

Lipopeptides from *Bacillus* are classified into three families according to the structure of the peptide moiety as follows: surfactins, iturins (mycosubtilins), and plipastatins/fengycins. These are amphiphilic cyclic peptides that are linked to a fatty acid hydrocarbon chain found to exert many biological effects. These molecules are largely reported not only for their biosurfactant capacities (especially surfactins) but also for their antimicrobial activities (Jacques, 2011). Several studies have revealed the potential of *Bacillus* strains as biosurfactant producers such as lipopeptides, as reported by Joshi et al. (2013) based on 77 isolates. Various studies reported the antifungal properties of the iturin- and plipastatin-families against food and plant pathogens (Ongena and Jacques, 2008; Kourmentza et al., 2021). Jemil et al. (2017a) revealed the antioxidant, antimicrobial, and anti-adhesive properties of the DCS1 lipopeptide from *B. methylotrophicus* DCS1. Recently, Abdollahi et al. (2020) revealed the antioxidant and anti-biofilm activities of surfactins from *B. amyloliquefaciens* NS6. In the field of medicine, surfactins are known for their antitumor, antiviral, anti-*Legionella*, and antiplatelet aggregation activities (Vollenbroich et al., 1997; Kim et al., 2006; Park and Kim, 2009; Vassaux et al., 2021) as well as anti-inflammatory properties (Tang et al., 2010). In the food industry, due to several techno-functional properties such as emulsifying, antibiofilming, and improving organoleptic properties of lipopeptides (Kiran et al., 2017), they are involved in the preservation of fruits and vegetables (Zhang et al., 2019). Moreover, the antioxidant properties, for example, wound healing activity (Ohadi et al., 2017) and anti-wrinkle/moisturizing activities (Kanlayavattanakul and Lourith, 2010) of lipopeptides, rhamnolipids, and glycolipids produced by bacterial strains are studied in some fields, such as cosmetics and food industries, for their capacity to prevent lipid oxidation, which is considered to be one of the major causes of quality deterioration in natural and processed foods (Hmidet et al., 2020).

Recently, Adeniji and Babalola (2019) revealed that an *in silico* analysis of the strain genome by antiSMASH could permit to predict which antioxidant molecules could potentially be produced by a strain. In their study, they showed that *Bacillus valezensis* NWUMFkBS10.5 was potentially able to produce antioxidant molecules such as lampranthin-2, miraxanthin V, and 2-decarboxybetanidin.

**Figure 1:**
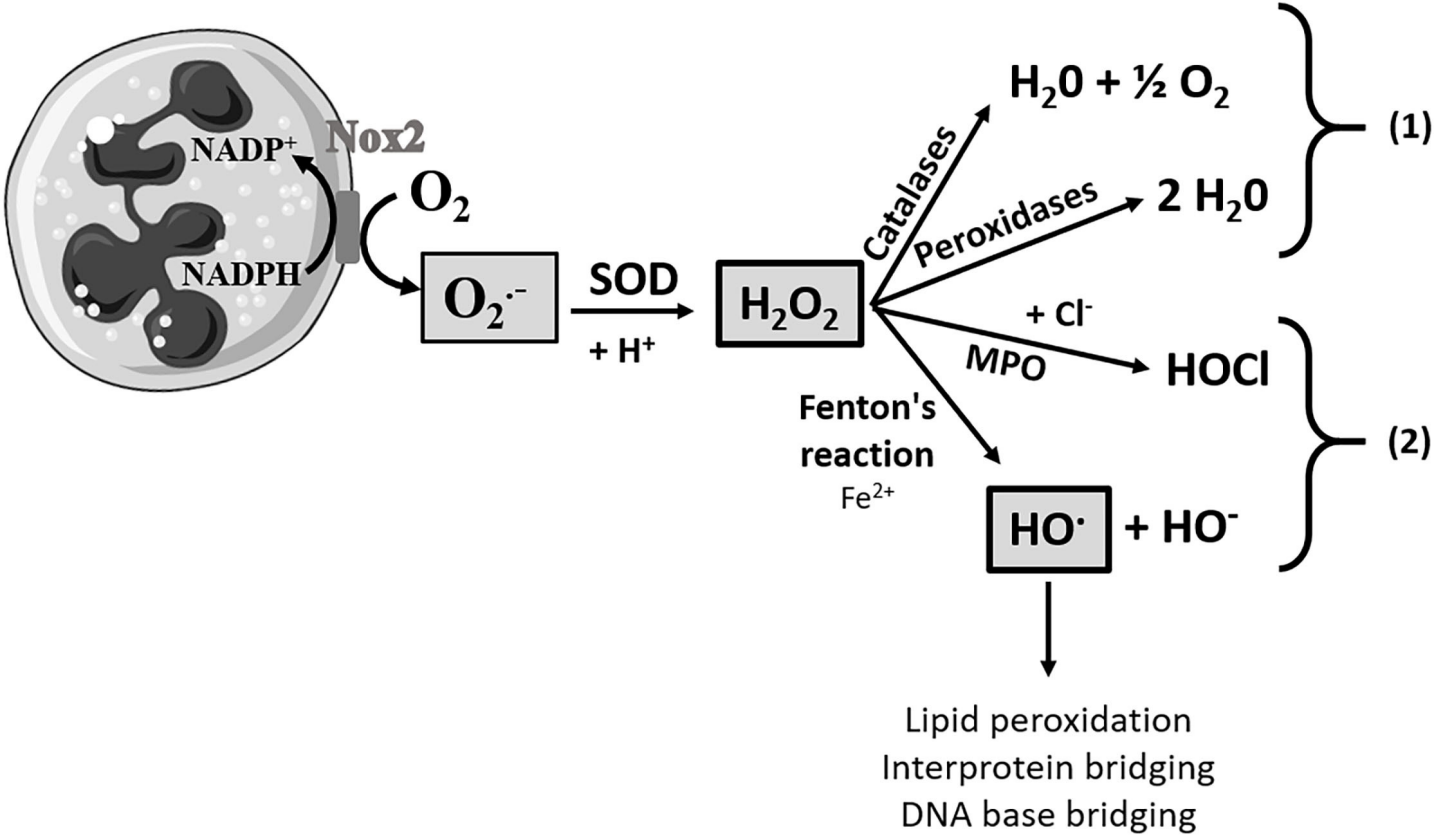
Pathways of formation of reactive oxygen species. Here is a neutrophilic polynuclear cell and an oxidative cascade. A large amount of the superoxide anion will be produced by the action of NADPH oxidase (Nox2). The production of the superoxide anion will be regulated by a detoxification enzyme, superoxide dismutase (SOD), and thus will be dismuted into hydrogen peroxide (H_2_O_2_). H_2_O_2_ can be protected by the antioxidant system composed of catalases and peroxidase, pathway (1). However, when this molecule is present in a too large quantity, the antioxidant system will be overwhelmed and the hydrogen peroxide will either be transformed into hypochlorous acid (HOCl) by myeloperoxidase (MPO) or it will be transformed into the hydroxyl radical (HO^.^), in the presence of Fe^2+^ by the Fenton reaction, pathway (2). HO^.^ is at the origin of lipid peroxidation, interprotein bridging, and DNA base bridging, and it is thus an extremely reactive and deleterious species for the tissues and cells.

The antioxidant activity of a (bio)chemical compound corresponds to its capacity to delay or prevent the oxidation of a substrate, resulting from an imbalance between ROS production and their degradation by antioxidants. As shown in Figure 1, in mammalian cells, ROS produced during the respiratory outbreak of macrophages, such as neutrophil granulocytes or lymphocytes, are essential actors in the initiation of the inflammatory process. Indeed, the oxidative cascade generates 3 essential ROS produced at different levels. The ${\text{O}}_{2}^{.-}$ species, which is the first element of the oxidative cascade, is formed from the addition of one electron to dioxygen and could be considered a primary ROS (Miller et al., 1990). The production of this ${\text{O}}_{2}^{.-}$ is regulated by the detoxification enzyme superoxide dismutase (SOD) and the ${\text{O}}_{2}^{.-}$ species is dismuted into H_2_O_2_ and dioxygen. H_2_O_2_ can be restored by different antioxidant systems (e.g., catalases and peroxidases like glutathione peroxidase) which can be overwhelmed in the case of an excess of H_2_O_2_. Finally, dreadful HO^.^, the neutral form of the hydroxide ion, can be produced by the Fenton reaction from H_2_O_2_ and in the presence of Fe^2+^. HO^.^ is the most reactive on the cellular membranes, making it a very dangerous radical (Pastor et al., 2000). In food industry, for example, some ROS such as hydroxyl radical and hydroperoxyl radical also initiate lipid peroxidation in meat, causing a rapid deterioration of meat lipids (Min and Ahn, 2005).

The aim of this study was to investigate, in a systemic way, the antiradical and antioxidant properties of the three families of lipopeptides (i.e., surfactins, mycosubtilins, and plipastatins) from *B. subtilis* strains using a conventional test, the ability of lipopeptides to act as free radical scavengers or hydrogen donors (inhibition of the 2,2-diphenyl-1-picrylhydrazyl (DPPH) radical), and three tests targeting the ROS generation by the oxidative cascade, the inhibition of ${\text{O}}_{2}^{.-}$, H_2_O_2_, and HO^.^. These cell-free pharmacological models, based on the spectrophotometric assay, were used in order to highlight the direct effects exerted by the lipopeptides on the different ROS generated during the oxidative cascade. Indeed, the method using DPPH, a stable free radical, is one of the most common antioxidant assays, allowing the evaluation of the radical quenching activity of compounds and has the advantage of being unaffected by side reactions, such as metal chelation and/or enzyme inhibition. However, the DPPH method focused on the radical scavenging capacity of the molecules, without any evaluation of their potential ability to enhance the function of endogenous antioxidant enzymes at different levels of the oxidative cascade and target ROS specifically. The use of a common test DPPH and also specific tests targeting ROS such as ${\text{O}}_{2}^{.-}$ and HO^.^, two oxygen-derived free radical species, and H_2_O_2_ (non-radical species) is essential to study the free radical-scavenging activity of *Bacillus*'s lipopeptides and fully characterize their antiradical/antioxidant effects. This comprehensive approach has been widely used for other compounds such as borage and evening primrose extracts (Wettasinghe and Shahidi, 2000), fruits extracts (Hazra et al., 2010), and exopolysaccharides (Li et al., 2014), but never for lipopeptides produced by *B. subtilis*.

## Materials and Methods

### Lipopeptide Production and Characterization

Surfactins (sodium salt) were supplied by Kaneka (Kenaka Co. Ltd., Japan). This molecule was most likely produced by *B. subtilis* SD901 (FERM BP-7666), as described in the patent WO2012/043800 (Nakayama et al., 1997). Mycosubtilins were produced by the strain *B. subtilis* LBS1, purified, and kindly provided by Lipofabrik (Villeneuve d'Ascq, France). Plipastatins were produced by the strain *B. subtilis* Bs2504, purified, and kindly provided by Lipofabrik (Villeneuve d'Ascq, France). Details of the culture conditions have been recently reported by Kourmentza et al. (2021). Lipopeptides were solubilized at 1 g.L^−1^ in methanol (MeOH) and the isoform composition was characterized by reverse phase-high pressure liquid chromatography (RP-HPLC) using an Acquity H-Class (Waters, Massachussetts, USA) coupled with a photodiode array (PDA) and an Acquity QDa mass spectrometer (Waters). The separation was performed using an Interchim Uptisphere TP 300Å C18 (250 × 3.0 mm, 5 μm) column. The solvents used for the separation were (A) MilliQ water with 0.1% formic acid and (B) acetonitrile (HPLC grade) with 0.1% formic acid. The solvent flow rate was set at 0.6 ml/min. The solvent gradient was set as follows: start −95% A/5% B from 0 to 5 min; from 5 to 40 min – from 95% A/5% B to 0% A/100% B; from 40 to 45 min – 0% A/100% B; from 45 to 46 min – from 0% A/100% B to 95% A/5% B; from 46 to 56 min – 95% A/5% B. Compound ionization and mass over charge (m/z) ratio measurements were performed in the positive mode with a heated electrospray (HESI)-Acquity QDa mass spectrometer. The ion source was set at a voltage of 15 kV and a desolvation temperature of 600°C and the m/z range 200–1,250 was used for the mass spectrometry (MS) measurements. The lipopeptide isoforms were identified according to their molecular mass. The lipopeptide quantification was carried out using the absorbance at 214 nm compared with the standard solutions of surfactins A, fengycins, and iturins A which were purchased from Sigma-Aldrich (Sigma-Aldrich, St. Louis, MO, USA).

### Antiradical and Antioxidant Activities of Lipopeptides

To study the free radical scavenging and antioxidant properties of the three families of lipopeptides (i.e., surfactins, mycosubtilins, and plipastatins) from *B. subtilis* strains, a conventional test to determine the ability of lipopeptides to act as free radical scavengers or hydrogen donors (inhibition of the 2,2-diphenyl-1-picrylhydrazyl radical (DPPH)), and three tests targeting ROS generated at different stages of the oxidative cascade, ${\text{O}}_{2}^{.-}$, H_2_O_2_, and HO^.^ (Figure 1), were performed.

#### Free Radical Scavenging by the Use of the DPPH Radical

The DPPH radical scavenging capacity of each lipopeptide was determined according to the method of Galasso et al. (2020). Various concentrations of the compounds (surfactins, mycosubtilins, plipastatins, and the mixture of surfactins/mycosubtilins and surfactins/plipastatins) i.e., 100, 200, 300, 400, and 500 mg.L^−1^ of each lipopeptide (for the mixture, the indicated concentrations corresponded to the concentration of each of the two lipopeptides) and of trolox as positive control, i.e., 0.98, 1.95, 3.91 and 7.81 mg.L^−1^ were tested for the radical scavenging assay. A volume of 50 μl of the samples or control were mixed in a 96-well plate with 150 μl of DPPH solution at a final concentration of 100 μM in MeOH (prepared daily), and allowed to react for 30 min in the dark and at room temperature. The diluents, methanol solution, and 1% dimethyl sulfoxide (DMSO) were used as a negative control for trolox and samples, respectively. At the end of the incubation, the absorbance was measured at 517 nm against blank controls containing the samples or trolox and MeOH (DPPH diluent), in order to avoid the absorbance of the samples, using a microplate reader (SpectraMax^®^, Molecular Devices, San José, USA). The experiment was carried out in quadruplicate. The results are presented as a percentage of DPPH inhibition with respect to the MeOH/DMSO negative control. Radical scavenging activity was calculated using the following formula: % inhibition = [(AC - AS)/AC] × 100 with AC, absorbance of the negative control, and AS, absorbance of the samples.

Half maximal inhibitory concentration (IC_50_) values were determined from the results obtained. IC_50_ denotes the concentration of the sample/drug required to scavenge 50% of the DPPH free radicals compared to the control without a sample.

#### In vitro Measurement of the Antioxidative Effect of the Lipopeptides on the Superoxide Anion

The ${\text{O}}_{2}^{.-}$ inhibition by different lipopeptides (e.g., surfactins, mycosubtilins, plipastatins, surfactins/plipastatins, and surfactins/mycosubtilins mixture) was quantified according to Aruoma et al. (1989) using the reduction of ferricytochrome C. The ${\text{O}}_{2}^{.-}$ (in the range of 8–12 μmol.L^−1^) was produced in Hank's-HEPES (HH) buffered medium at pH 7.42 in each assay tube using the biochemical system xanthine (0.1 mmol.L^−1^)/xanthine oxidase (50 mU.ml^−1^) or X/XO. The samples in predefined increasing concentrations (62.5, 125, 187.5, and 250 mg.L^−1^ of lipopeptide or each of the two lipopeptides for the mixture) were brought into contact with the required amount of ${\text{O}}_{2}^{.-}$ for 15 min at a temperature of 25°C in the presence of equine ferricytochrome C (0.017 mmol.L^−1^). The free radicals, not inhibited by the samples, reduced the ferricytochrome C, which changed from orange to pink colorand whose absorbance was read at a 550 nm wavelength using a Multiskan FC spectrophotometer (Thermo Fisher Scientific Instruments Co, Shanghai, China) against blank controls containing all the reagents except X/XO, in order to avoid the absorbance of the samples or solutions during spectrophotometry. Negative controls without the sample containing only ${\text{O}}_{2}^{.-}$ and positive controls of ${\text{O}}_{2}^{.-}$ inhibition controls containing cysteine (Cys) (0.3 mmol.L^−1^) were also assessed in each series of tests. Finally, the ferricytochrome C extinction coefficient (ε550 nm = 2.11 × 10^−8^ M^−1^.cm^−1^) was used to convert the absorbances to nanomoles of the superoxide anion.

#### In vitro Measurement of the Antioxidative Effect of the Lipopeptides on Hydrogen Peroxide

The production of H_2_O_2_ was adopted from the method developed by Thurman et al. (1972) and Hochart-Behra et al. (2014) using the absorption at a wavelength (λ) of 480 nm of red ferrithiocyanate complexes formed in the presence of peroxides. In this cell-free model, H_2_O_2_ (approximately 13–15 μmol.L^−1^) was incubated at room temperature for 15 min with the samples (i.e., surfactins, mycosubtilins, plipastatins, surfactins/plipastatins, and surfactins/mycosubtilins mixture) of increasing concentrations (62.5, 125, 187.5, and 250 mg.L^−1^ of lipopeptide or each of the two lipopeptides for the mixture) in HH buffer solution at pH 7.42. The medium was then acidified with 40 μl of HNO_3_ (1 N). After addition of 200 μl of ammoniacal iron (II) sulfate (10 mol.L^−1^) and 100 μl of KSCN (2.5 mol.L^−1^) and vortexing, the absorbances of the media reaction were measured spectrophotometrically (λ_480_ nm). Blank controls containing all the reagents except H_2_O_2_ and positive inhibition controls of H_2_O_2_ were also examined with ascorbic acid (AA) (30 μmol.L^−1^) or Cys (0.3 mmol.L^−1^). Using a standard range with final H_2_O_2_ concentrations from 2.5 to 20 nmol.ml^−1^, it was possible to deduce H_2_O_2_ concentrations in nmol.ml^−1^ of the reaction medium.

#### In vitro Measurement of the Antioxidative Effect of the Lipopeptides on the Hydroxyl Radical

The inhibition of HO^.^ by the lipopeptides (surfactins, mycosubtilins, plipastatins, surfactins/plipastatins, and surfactins/mycosubtilins mixture) was evaluated according to a method adopted from Halliwell et al. (1987). In this model, HO^.^ was produced from 8 to 11 μmol.L^−1^ of H_2_O_2_ in each tube in buffered medium (20 mM KH_2_PO_4_ at pH 7.4) in the presence of FeCl_3_ 100 μmol.L^−1^, 104 μmol.L^−1^ EDTA, and 100 μmol.L^−1^ ascorbic acid to generate HO^.^ *via* Fenton's reaction. The presence of increasing concentrations (62.5, 125, 187.5, and 250 mg.L^−1^) of lipopeptide or each of the two lipopeptides for the mixture, with this HO^.^ resulted in an inhibition of HO^.^ in the case of the antioxidant effect toward this ROS. After the addition of deoxyribose (DR, 3 mmol.L^−1^), this sugar will degrade in proportion to the residual HO^.^. The tubes were incubated at 37°C for 30 min. The DR fragments were heated by boiling for 20 min to generate malondialdehyde (MDA) in the presence of thiobarbituric acid (14 mmol.L^−1^) in an acid medium (trichloroacetic acid, 147 mmol.L^−1^). In this assay, the absorbance was red against blank control containing all reagents except H_2_O_2_. Negative controls without the sample and positive inhibition controls (Cys, 0.3 mmol.L^−1^) were also assessed in each series of tests. Absorbance was measured by spectrophotometry at a wavelength of 532 nm. The evaluation of the HO^.^ concentration (nmol.ml^−1^) was deduced from a standard curve, obtained from the increasing amounts of H_2_O_2_ concentrations.

### Statistical Analysis

For pharmacological *in vitro* assays, the data were analyzed from four (DPPH radical analysis) and six (for ${\text{O}}_{2}^{.-}$, H_2_O_2_, and HO^.^) independent assays using ANOVA in the case of data normality and variance homogeneity both checked using the Graphpad Prism software. In the other cases, the Kruskal-Wallis test was used at the 5% level (*p* = 0.05) using the Graphpad Prism 8.0.1 software. Results were presented as a bar chart with means ± SD.

## Results

### Lipopeptide Characterization by LC-MS

Lipopeptides used in these studies were first characterized by RP-HPLC-PDA-MS analysis (Supplementary Figure S1). RP-HPLC-PDA-MS characterization of the surfactin solution revealed the presence of 4 surfactin isoforms displaying a m/z ([M+H]^+^) value of 994, 1008, 1022, and 1036 with a ratio of 7%, 24%, 35% and 24%, respectively, and corresponding to [Leu_7_ or Ile_7_] surfactin isoforms having either a C_12_-, or a C_13_-, or a C_14_-, or a C_15_-fatty acid chain and/or [Val_7_] surfactin isoforms having either a C_13_-, or a C_14_-, or a C_15_-, or a C_16_- fatty acid chain. These results were in accordance with those previously published by Taira et al. (2015). The mycosubtilin isoforms were 3% for the C_15_ isoforms (*n* and *iso*) ([M+H]^+^ = 1057), 52% for the C_16_ isoforms (*n* and *iso*) ([M+H]^+^ = 1071), 42% for the C_17_ isoforms (*iso* and *anteiso*) ([M+H]^+^ = 1085), and 3% for the C_18_ isoforms (*n* and *iso*) ([M+H]^+^ = 1099). These findings were in accordance with those published by Kourmentza et al. (2021). RP-HPLC-PDA-MS analysis of the solubilized plipastatins revealed numerous isoforms. The following [M+2H]^2+^ values of plipastatin and their respective ratio were 724 (2%), 731 (1%), 732 (6%), 738 (15%), 739 (27%), 745 (7%), 746 (34%), 753 (2%), and 760 (1%), corresponding to the plipastatin A and B isoforms with fatty acid chains between C_14_ and C_19_. Among these plipastatin isoforms, three ([M+2H]^2+^ = 731 (1%), 738 (15%), and 745 (7%) have a monounsaturated fatty acid chain. So, the most abundant (83%) isoforms in this mixture were the saturated C_17_ plipastatin A ([M+2H]^2+^ = 739 (27%)), the monounsaturated C_15_ plipastatin B ([M+2H]^2+^ = 738 (15%)), the saturated C_18_ plipastatin A ([M+2H]^2+^ = 746 (34%), and the monounsaturated C_16_ plipastatin B ([M+2H]^2+^ = 745 (7%). These results were in accordance with those published by Hamley et al. (2013).

### DPPH Scavenging Activity

**Figure 2:**
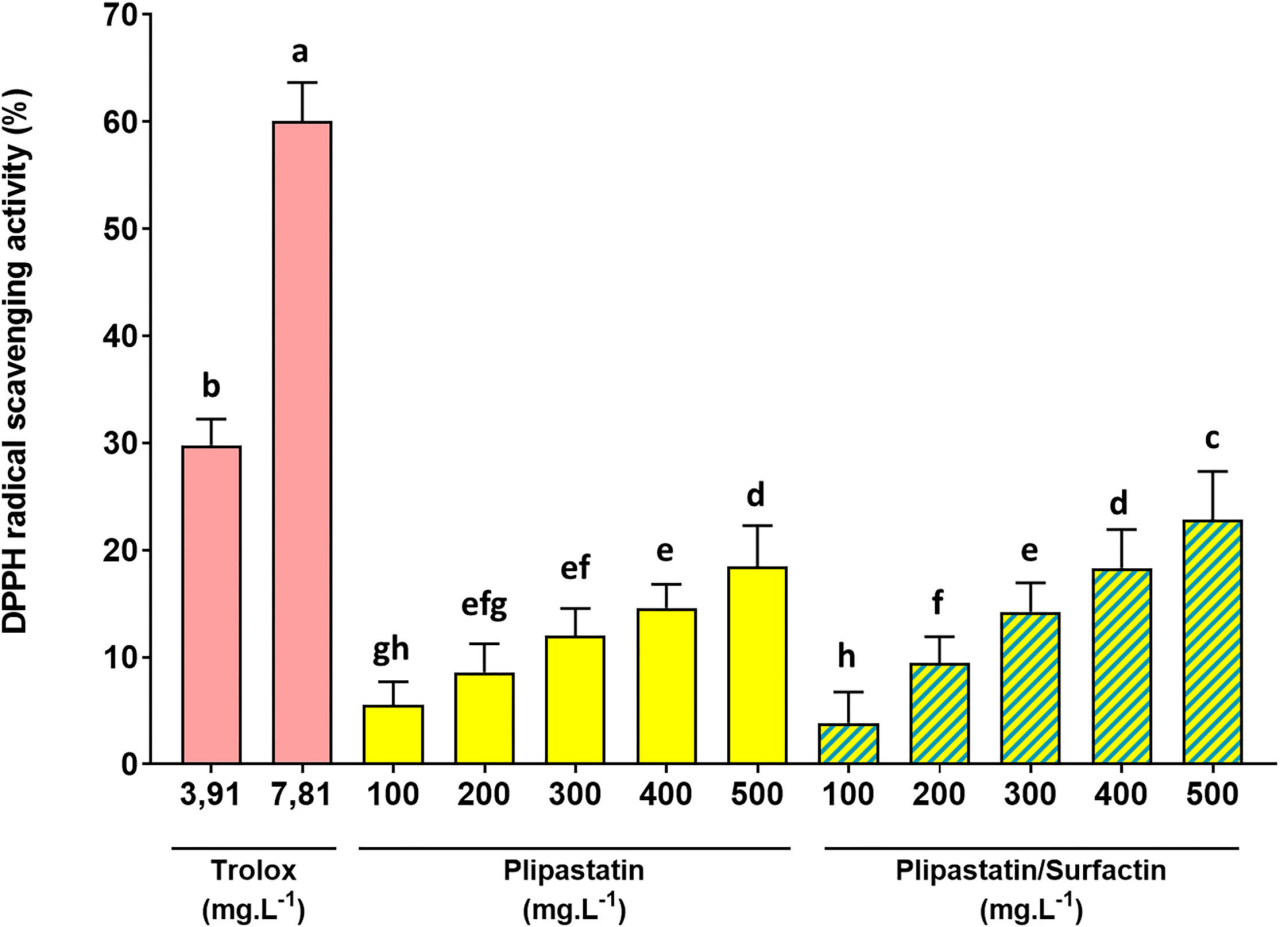
*In vitro* inhibition of the DPPH radical by the lipopeptides of interest at increasing concentrations. The effects on the DPPH radical of 100 to 500 mg.L^−1^ of the compounds were compared (for the mixture, the indicated concentrations correspond to the concentration of each of the two lipopeptides). Trolox was used as the positive control. The DPPH radical scavenging activity was expressed as % of the negative control. Statistical analysis of the data was performed on 4 independent experiments using ANOVA (overall Fisher's test at the *p* = 0.05 level) and the Tukey's multiple *a posteriori* comparison test with the Graph Pad Prism software. Means without a common letter (a-h) are different (*p* < 0.05). Results are presented as a bar chart with means (*n* = 16) ± SD.

As displayed in Figure 2, trolox (positive control) exhibited a remarkable ability to inhibit the DPPH radical. A concentration range of trolox was analyzed in order to calculate the IC_50_ values and validate the test (data not shown). The concentration of 3.91 mg.L^−1^ of trolox showed a statistically significant inhibition of the DPPH radical compared to the negative control. Indeed, the percentage of inhibition increased from 0 ± 1.19 (MeOH negative control) to 29.80 ± 2.45% for the trolox concentration of 3.91 mg.L^−1^ and the percentage of inhibition reached 60.04 ± 3.63% (Dunn's multiple comparison test, *p* < 0.0001) for the trolox concentration of 7.81 mg.L^−1^. IC_50_ could be calculated for trolox and the concentration range tested (6.29 mg.L^−1^).

Plipastatins and mixture of surfactins/plipastatins also exhibited an effective antiradical activity against DPPH in a dose-dependent manner. For plipastatins, this effect compared with that of the control without lipopeptide was statistically significant (*p* = 0.0023, using the Dunn's multiple comparison test) for 300 mg.L^−1^ of the sample and extremely significant (*p* < 0.0001) for the higher concentration (500 mg.L^−1^) of plipastatins. Plipastatins showed a potential scavenging effect of 18.48 ± 3.83% at 500 mg.L^−1^. The inhibition of the DPPH radical by the mixture of surfactins/plipastatins was also statistically significant (*p* < 0.0001, using the Dunn's test) for 300 mg.L^−1^ of the sample. The mean inhibition of the DPPH radical by the mixture of surfactins/plipastatins reached 22.88 ± 4.47% at the highest concentration of the mixture tested (500 mg.L^−1^). Concomitantly, no inhibitory effect was observed for surfactins, mycosubtilins, and the mycosubtilins/surfactins mixture.

However, as shown in Figure 2, plipastatins and surfactins/plipastatins mixture exhibited lower radical-scavenging activity than trolox, used as a reference; the inhibition obtained for each of the concentrations was not statistically comparable to the inhibition obtained for a concentration of 3.91 mg.L^−1^ of trolox (ANOVA, Tukey's multiple comparison test).

### Superoxide Anion Inhibition

**Figure 3:**
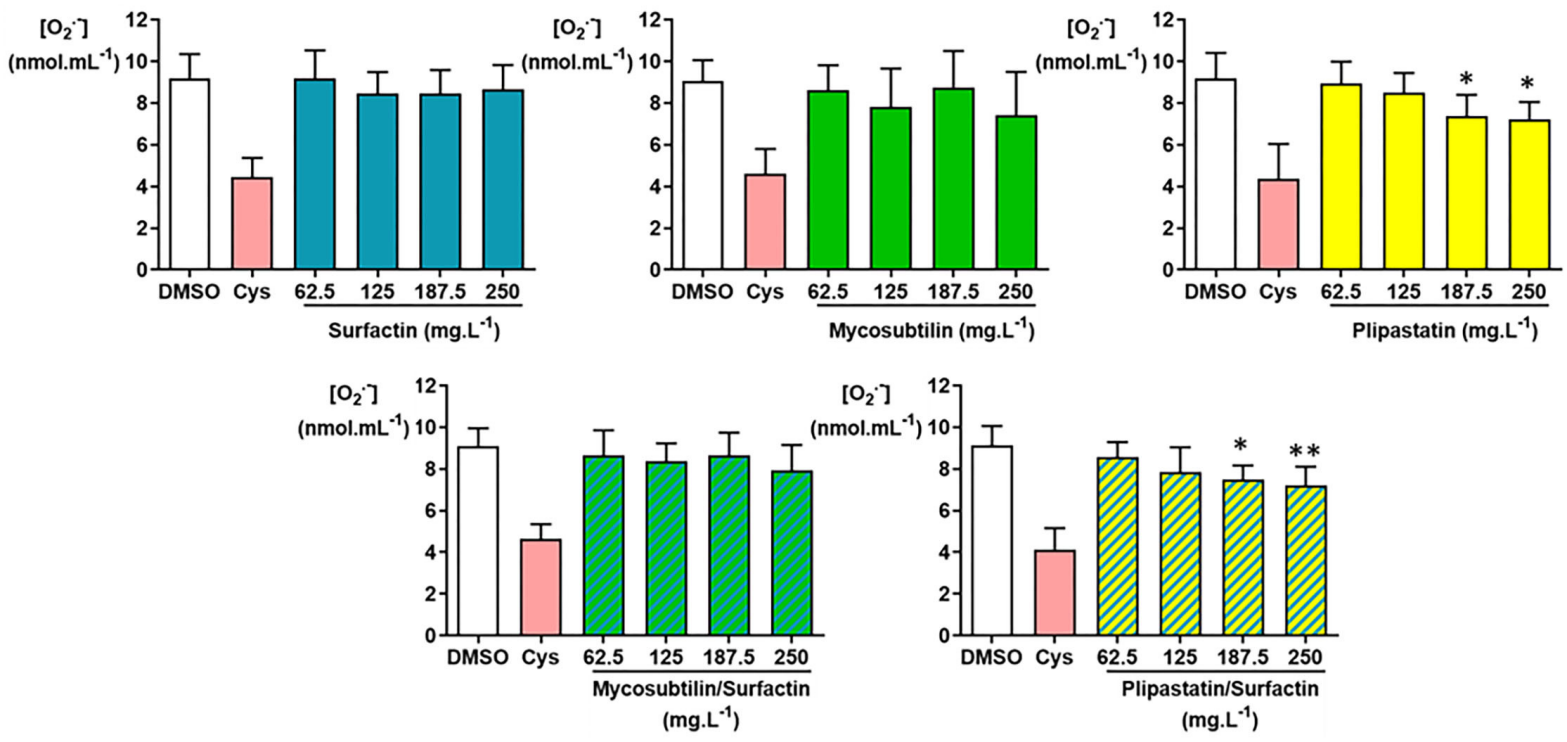
Inhibition of the superoxide anion (${\text{O}}_{2}^{.-}$) by the following lipopeptides (surfactin, mycosubtilin, plipastatin, mixture of mycosubtilin/surfactin, and mixture of plipastatin/surfactin) at increasing concentrations in a cell-free model. The effects on ${\text{O}}_{2}^{.-}$ of lipopeptides tested at concentrations between 62.5 and 250 mg.L^−1^ (for the mixture, the indicated concentrations correspond to the concentration of each of the two lipopeptides) were compared to those of the DMSO-negative control. The positive control for ${\text{O}}_{2}^{.-}$ inhibition was cysteine (Cys) at 0.3 mmol.L^−1^. Statistical analysis of the data was performed on 6 independent experiments using ANOVA (overall Fisher's test at the *p* = 0.05 level and Tukey's multiple *a posteriori* comparison test) for mycosubtilin, plipastatin, mycosubtilin/surfactin, and plipastatin/surfactin and using the Kruskal-Wallis test (Dunn's multiple *a posteriori* comparison test) for surfactin, with the GraphPad Prism software, *p < 0.05, **p < 0.01). Results are presented as a bar graph (means ± SD).

As shown in Figure 3, the ${\text{O}}_{2}^{.-}$-inhibiting concentrations of negative- (DMSO) and positive (Cys)-controls were as expected. Therefore, only plipastatins and plipastatins/surfactins mixture showed a statistically significant *in vitro* inhibition of the superoxide anion using one-way ANOVA (*p* = 0.007 and *p* = 0.0058, respectively (with the overall Fisher's test at the *p* = 0.05 level)). Compared with the non-inhibition value of the DMSO-negative control, the plipastatin inhibition was statistically significant for the lipopeptide concentrations of 187.5 mg.L^−1^ (*p* = 0.0411 using the Tukey's multiple comparison test) and 250 mg.L^−1^ (*p* = 0.023). The mean concentration of the superoxide anion decreased from 9.18 ± 0.12 nmol.ml^−1^ for the control to 7.24 ± 0.81 nmol.ml^−1^ at 250 mg.L^−1^ of plipastatins, corresponding to a 21% inhibition of ${\text{O}}_{2}^{.-}$ production.

The inhibition of ${\text{O}}_{2}^{.-}$ by the mixture of plipastatins/surfactins was also statistically significant for 187.5 mg.L^−1^ of the mixture (*p* = 0.0292 using the Tukey's test). The mean difference between the ${\text{O}}_{2}^{.-}$ concentration obtained for the DMSO-negative control and those observed for 250 mg.L^−1^ of mixture reached about 1.94 nmol.ml^−1^ of ${\text{O}}_{2}^{.-}$, corresponding to a 21% inhibition of ${\text{O}}_{2}^{.-}$ production, as this ${\text{O}}_{2}^{.-}$ concentration decreased from 9.14 ± 0.93 to 7.20 ± 0.90 nmol.ml^−1^ (*p* = 0.0082).

The inhibition of ${\text{O}}_{2}^{.-}$ production by surfactins, mycosubtilins, and the mycosubtilin/surfactin mixture achieved about 6, 19, and 13%, respectively, at the highest concentration of lipopeptides tested (250 mg.L^−1^), as the mean ${\text{O}}_{2}^{.-}$ concentration decreased from 9.19 ± 1.16 (DMSO-negative control) to 8.65 ± 1.18 nmol.ml^−1^ for the concentration of 250 mg.L^−1^ of surfactin, from 9.06 ± 0.99 (DMSO-negative control) to 7.43 ± 2.07 nmol.ml^−1^ for the concentration of 250 mg.L^−1^ of mycosubtilins, and from 9.11 ± 0.85 (DMSO-negative control) to 7.92 ± 1.22 nmol.ml^−1^ for the concentration of 250 mg.L^−1^ of the mycosubtilin/surfactin mixture. However, these results showed no statistically significant differences.

### Hydrogen Peroxide Inhibition

**Figure 4:**
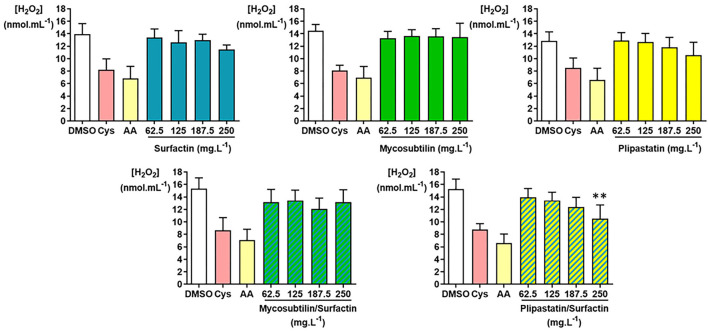
Inhibition of hydrogen peroxide (H_2_O_2_) by the lipopeptides of interest (surfactin, mycosubtilin, plipastatin, mixture of mycosubtilin/surfactin, and mixture of plipastatin/surfactin) in a cell-free model. The effects on H_2_O_2_ of lipopeptides tested at concentrations between 62.5 and 250 mg.L^−1^ (for the mixture, the concentrations indicated correspond to the concentration of each of the two lipopeptides) were compared to those of the DMSO-negative control. The positive controls for H_2_O_2_ inhibition were cysteine (Cys) and ascorbic acid (AA) tested at concentrations 0.3 mmol.L^−1^ and 30 μmol.L^−1^, respectively. Statistical analysis of the data was performed with 6 independent experiments using ANOVA (overall Fisher's test at the *p* = 0.05 level and Tukey's multiple *a posteriori* comparison test) for surfactin, mycosubtilin, plipastatin, and mycosubtilin/surfactin and using the Kruskal-Wallis test (Dunn's multiple *a posteriori* comparison test) for plipastatin/surfactin, with the GraphPad Prism software, **p < 0.01). Results are presented as a bar graph (means ± SD).

As presented in Figure 4, the inhibition or non-inhibition of the H_2_O_2_ production was as expected for the DMSO-negative control and the cysteine (Cys)- and acid ascorbic (AA)-positive controls. Only the plipastatin/surfactin mixture showed a statistically significant *in vitro* inhibition of H_2_O_2_ using the Kruskal-Wallis test (*p* = 0.0053). This inhibition level compared with those of the DMSO-negative control was statistically significant for 250 mg.L^−1^ (*p* = 0.0040 using the Dunn's multiple comparison test). The mean concentration of H_2_O_2_ decreased from 15.25 ± 1.61 nmol.ml^−1^ for the DMSO-negative control to 10.53 ± 2.19 nmol.ml^−1^ at 250 mg.L^−1^ of plipastatin/surfactin mixture, corresponding to 31% inhibition of H_2_O_2_ production.

The inhibition of H_2_O_2_ production by surfactins reached about 21% at the highest concentration of lipopeptide tested (250 mg.L^−1^), as the mean H_2_O_2_ concentration diminished from about 13.94 ± 1.68 (DMSO-negative control) to 11.45 ± 0.75 nmol.ml^−1^ for the concentration of 250 mg.L^−1^ of surfactin. However, this result showed no statistically significant differences. Concerning the inhibition of H_2_O_2_ production by mycosubtilins, plipastatins, and mixture of mycosubtilins/surfactins, it reached, at the highest concentration of lipopeptides tested (250 mg.L^−1^), about 9, 18, and 16%, respectively.

### Hydroxyl Radical Inhibition

**Figure 5:**
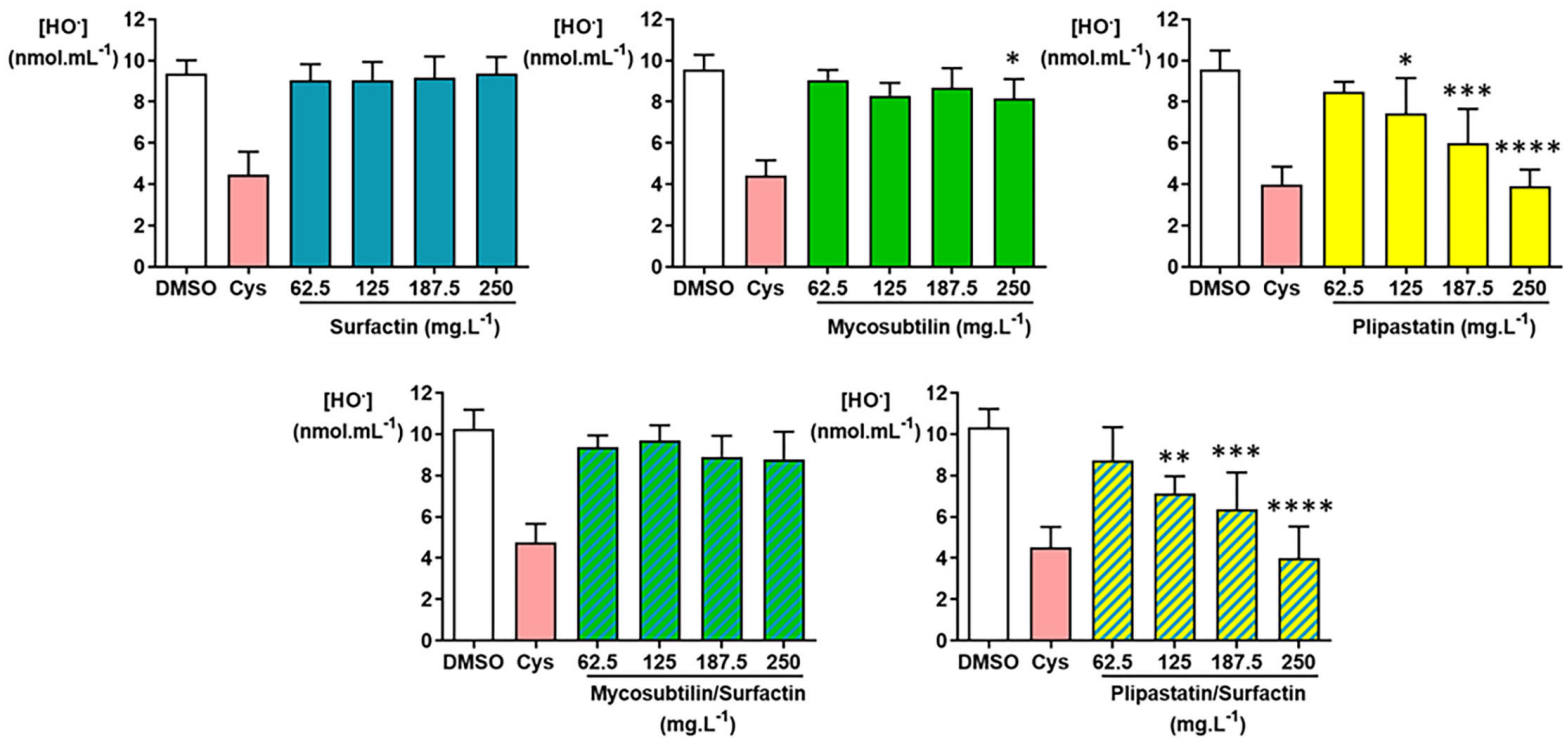
Inhibition of the hydroxyl radical (HO^.^) by the lipopeptides (surfactin, mycosubtilin, plipastatin, mixture of mycosubtilin/surfactin, and mixture of plipastatin/surfactin) in a cell-free model. The effects on HO^.^ of lipopeptides tested at concentrations between 62.5 and 250 mg.L^−1^ (for the mixture, the indicated concentrations correspond to the concentration of each of the two lipopeptides) were compared to those of the DMSO-negative control. The positive control for HO^.^ inhibition was cysteine (Cys) tested at 0.3 mmol.L^−1^. Statistical analysis of the data was performed with 6 independent experiments using ANOVA (overall Fisher's test at the *p* = 0.05 level and Tukey's multiple *a posteriori* comparison test) for mycosubtilin, plipastatin, mycosubtilin/surfactin, and plipastatin/surfactin and using the Kruskal-Wallis test (Dunn's multiple *a posteriori* comparison test) for surfactin, with the GraphPad Prism software, *p < 0.05, **p < 0.01, ***p < 0.001, ****p < 0.0001. Results are presented as a bar graph (means ± SD).

The inhibition or non-inhibition of the HO^.^ production was as expected for the DMSO-negative control and the cysteine (Cys)-positive controls (Figure 5) and 6 independent experiments were carried out. As shown in Figure 5, plipastatins and the plipastatin/surfactin mixture showed strong inhibitory effects against HO^.^. These inhibitions were statistically significant (overall Fisher's test, *p* < 0.0001) at the concentration of 125 mg.L^−1^ for plipastatins and the plipastatin/surfactin mixture (*p* = 0.0443 and 0.0040, respectively) using the Tukey's multiple comparison test.

The decreases of the HO^.^ mean concentration were remarkable for both lipopeptides tested at 250 mg.L^−1^ compared with the DMSO-negative control, i.e., from 9.55 ± 0.93 to 3.90 ± 0.81 nmol.ml^−1^ of HO^.^ for plipastatins (*p* < 0.0001) and from 10.33 ± 0.90 to 4.00 ± 1.52 nmol.ml^−1^ of HO^.^ for the plipastatin/surfactin mixture (*p* < 0.0001), corresponding to 59 and 61% inhibition of HO^.^ production, respectively.

A lesser but statistically significant inhibitory effect of mycosubtilins was also observed against HO^.^ from the concentration of 250 mg.L^−1^ using ANOVA (overall Fisher's test, *p* = 0.0215; Tukey's multiple comparison test, *p* = 0.0261) where the HO^.^ concentration decreased from 9.59 ± 0.69 nmol.ml^−1^ of HO^.^ (DMSO-negative control) to 8.17 ± 0.94 nmol.ml^−1^ of HO^.^ at 250 mg.L^−1^ of mycosubtilins. In the same way, the inhibition of HO^.^ production by the surfactins and the mycosubtilin/surfactin mixture reached, at the highest concentration of lipopeptides tested (250 mg.L^−1^) (not significant), about 3 and 15%, respectively. The calculated IC_50_ value was 222.5 mg.L^−1^ for both the plipastatin and plipastatin/surfactin mixture.

## Discussion

The aim of our study was to evaluate the antiradical and antioxidant properties of three lipopeptide families (i.e., surfactin, mycosubtilin, and plipastatin) produced by *Bacillus subtilis* strains, using several *in vitro* tests (inhibition of DPPH, ${\text{O}}_{2}^{.-}$, H_2_O_2_, and HO^.^), more or less specific of the ROS produced by the oxidative cascade. Concerning the DPPH inhibition test, the results obtained for the trolox positive control (IC_50_ = 6.29 mg.L^−1^, corresponding to 25.1 μM) were very close to those reported by Alfieri et al. (2020) (6 mg.L^−1^, corresponding to 23.97 μM) and Tabbene et al. (2012) (23 μM), validating the obtained data and the use of this test.

Our results showed that among the three lipopeptides tested, plipastatins (alone or mixed with surfactins) have the strongest antiradical and antioxidant effects. Plipastatins significantly decreased DPPH (from 300 mg.L^−1^), ${\text{O}}_{2}^{.-}$ (from 187.5 mg.L^−1^), and HO^.^ production (from 125 mg.L^−1^), in a dose-dependent manner. With the DPPH scavenging activity, we achieved about 20% inhibition for plipastatins and the surfactin/plipastatin mixture at the highest concentration tested (500 mg.L^−1^). The tested lipopeptides exhibited a lower radical-scavenging activity than that was previously reported for other lipopeptides. Indeed, Jemil et al. (2017a) showed that the DCS1 lipopeptides from *B. methylotrophicus* DCS1 exhibited a potential scavenging effect of 25.9% at 100 mg.L^−1^ and of 80.6% at 1,000 mg.L^−1^. The lipopeptides produced by the strain *B. methylotrophicus* DCS1 have been described in another study and correspond to a mixture containing four isoforms of surfactin, four isoforms of pumilacidin, five isoforms of iturin A, five isoforms of bacillomycin D, and six isoforms of fengycin (Jemil et al., 2017b). In both studies the authors do not demonstrate which lipopeptides of this mixture are responsible for the activity. Nevertheless, its existence in the mixture of surfactin and fengycin is a common feature of our results illustrated in Figure 2. It was also reported that the mixture of lipopeptides isolated from *B. subtilis* VSG4 exhibited 69.1% DPPH radical scavenging activity at a concentration of 5,000 mg.L^−1^ (Giri et al., 2019). In another study, Ben Ayed et al. (2015) showed that the A21 lipopeptides exhibited a potential scavenging effect of 65% at 1,000 mg.L^−1^ and of 12.4% at 50 mg.L^−1^. It is noted that **i**) the concentrations tested in these studies were much higher than the concentrations we used, which may explain why IC_50s_ could not be calculated in our case, and **ii**) all these studies concerned mixtures of lipopeptides not necessarily identified. Indeed, Ben Ayed et al. showed that lipopeptides produced by *B. amyloliquefaciens* allowed 80% inhibition of DPPH at the concentration of 750 mg.L^−1^ (IC_50_ at 370 mg.L^−1^). However, the samples tested in this study contained surfactins, fengycins, and bacillomycins (Ben Ayed et al., 2017). We could therefore hypothesize that the DPPH scavenging effect of lipopeptides tested in this study was weaker because the lipopeptides were partially purified and not a very heterogeneous lipopeptide mixture. Finally, Yalçin and Çavuşoglu (2010), who studied surfactin-like molecules produced by *B. subtilis* RW-I, have reported an IC_50_ value estimated at 250 mg.L^−1^ for DPPH scavenging activity. In our study, surfactins did not show any inhibitory effect on DPPH up to 500 mg.L^−1^. This discrepancy may be due to the different protocols used, in particular the DPPH concentration and the DPPH/sample ratio. Moreover, the *Bacillus* genus is known for its production of other metabolites with antioxidant activity, for example phenolic and benzoic acids (Safronova et al., 2021) or even exopolysaccharides like levan (Pei et al., 2020). Therefore, the lipopeptide purification allows one to avoid the interference of other metabolites displaying the same inhibition activities.

Concerning the H_2_O_2_ inhibition, only the surfactin/plipastatin mixture showed a statistically relevant decrease in the production of this ROS. We could suggest that this effect was due to the combined properties of surfactins and plipastatins since neither surfactin alone nor plipastatins alone inhibited the H_2_O_2_ production in a significant manner.

The inhibition of HO^.^ by plipastatins and the surfactin/plipastatin mixture was observed. For the higher concentration tested (250 mg.L^−1^), the obtained inhibition was similar to those obtained for the Cys-positive control. This result is of prime importance because HO^.^ is a highly reactive and harmful species toward tissues (initiation of lipid peroxidation, cell membrane damage, and DNA destruction). The IC_50_ values obtained for both plipastatins and surfactin/plipastatin mixture were about 222.5 mg.L^−1^, which is more than 10-fold lower than the IC_50_ value obtained for the HO^.^ scavenging effect by Giri et al. (2019) (around 3,200 mg.L^−1^ of BS-VSG4). In addition, mycosubtilins showed a weak HO^.^ inhibitory effect.

In previous studies, the antioxidant activities of surfactins and fengycins/plipastatins were demonstrated. Wang et al. (2021) showed the antioxidant property of surfactins in an *in vivo* model (Zebrafish), by measuring the levels of superoxide dismutase, malondialdehyde, and glutathione peroxidase. In tomato, fengycins were demonstrated to be an antioxidant by inducing the accumulation of ROS in *Sclerotinia sclerotiorum* mycelium and downregulating the expression of ROS-scavenging genes compared to the negative control, thus reducing dramatically the lesion size (Farzand et al., 2019).

Furthermore, Yalçin and Çavuşoglu (2010) demonstrated that the reduction capacity of lipopeptides biosurfactants, regarding DPPH activity may be related to the presence of hydroxyl groups in their molecular structure. In addition, Tabbene et al. (2012) have shown that the antioxidant potential of the bacillomycin D could be related to the presence of tyrosine and proline residues in the peptide ring and the hydrocarbon fatty acid chain. Indeed, tyrosine residue *via* its phenolic hydroxyl group could transfer a proton to electron-deficient radicals and proline, and due to its pyrrolidine ring, it plays an important role in radical scavenging activity (Chen et al., 1996; Rajapakse et al., 2005). Concerning ${\text{O}}_{2}^{.-}$ and HO^.^, Tabbene et al. (2012) have suggested that these scavenging activity could also be linked to the presence of hydrophobic and aromatic residues as tyrosine and proline.

These proposed mechanisms of action, based on a molecular structure, were consistent with our results since plipastatin which contained two hydroxyl groups, two tyrosines, and one proline, was the most antiradical and antioxidant lipopeptide tested, regarding DPPH and ROS inhibitions (compared to surfactin which contained just two hydroxyl groups and mycosubtilin which contained one tyrosine and one proline).

## Conclusion

Considering the ${\text{O}}_{2}^{.-}$ production, the inhibition values obtained for the tested lipopeptides do not allow the calculation of IC_50_ since a relatively weak (5–20%) inhibitory effect was measured for the highest concentration tested (250 mg.L^−1^). Plipastatins and the plipastatin/surfactin mixture showed the best inhibitory activity (around of 20%) of the ${\text{O}}_{2}^{.-}$ production. For the mixture, this inhibitory activity was probably due to the presence of plipastatins. In contrast, surfactins, mycosubtilins, plipastatins, and the two tested mixtures did not show any significant inhibitory effect of the H_2_O_2_ production, except for plipastatins associated with surfactins (31% inhibition at the highest concentration tested). In contrast, when we focused on HO^.^, the last ROS in the oxidative cascade and the most reactive and dreaded ROS tested, a minimal inhibitory effect of mycosubtilins (only for the concentration of 250 mg.L^−1^) was observed. As for plipastatins and the plipastatin/surfactin mixture, the effects were more important for a calculated IC_50_ value of 222.5 mg.L^−1^. The other samples had no effect on this ROS. In conclusion, due to their antioxidant capacity, pure plipastatins and plipastatins mixed with surfactins were the best candidates for food and pharmaceutical applications.

## Data Availability Statement

The original contributions presented in the study are included in the article/Supplementary Material, further inquiries can be directed to the corresponding author.

## Author Contributions

ED, JB-M, RR, and FC conceptualized the experiments. ED, MT, AN, and CD took part in the investigation and methodology analysis. ED wrote the manuscript. CF, JB-M, RR, and FC helped in the critical review and the editing of the manuscript. RR and FC participated in the funding acquisition. All authors contributed to the article and approved the submitted version.

## Funding

This study was supported by the project BioSMART – Bio-based smart packaging for enhanced preservation of food quality – Grant agreement No. 745762, funded by the Bio-based Industries Joint Undertaking (BBI-JU) under the European Union's Horizon 2020 Research and Innovation Programme. This study was also supported by the University of Lille through the ALIBIOTECH program funding administered by the Hauts-de-France Region.

## Conflict of Interest

FC from the University of Lille is also the co-founder of Lipofabrik company which markets lipopeptides from *B. subtilis*. AN is also part of Lipofabrik company. The remaining authors declare that the research was conducted in the absence of any commercial or financial relationships that could be construed as a potential conflict of interest.

## Publisher's Note

All claims expressed in this article are solely those of the authors and do not necessarily represent those of their affiliated organizations, or those of the publisher, the editors and the reviewers. Any product that may be evaluated in this article, or claim that may be made by its manufacturer, is not guaranteed or endorsed by the publisher.
