# Supplementary material for: Evaluation of Antiradical and Antioxidant Activities of Lipopeptides Produced by Bacillus subtilis Strains
Source: Front Microbiol. 2022 Jun 20;13:914713. doi: 10.3389/fmicb.2022.914713 (PMC9251515; doi:10.3389/fmicb.2022.914713)
Supplement: Supplementary file 1 [file Table_1.DOCX]

**Supplementary data**

**
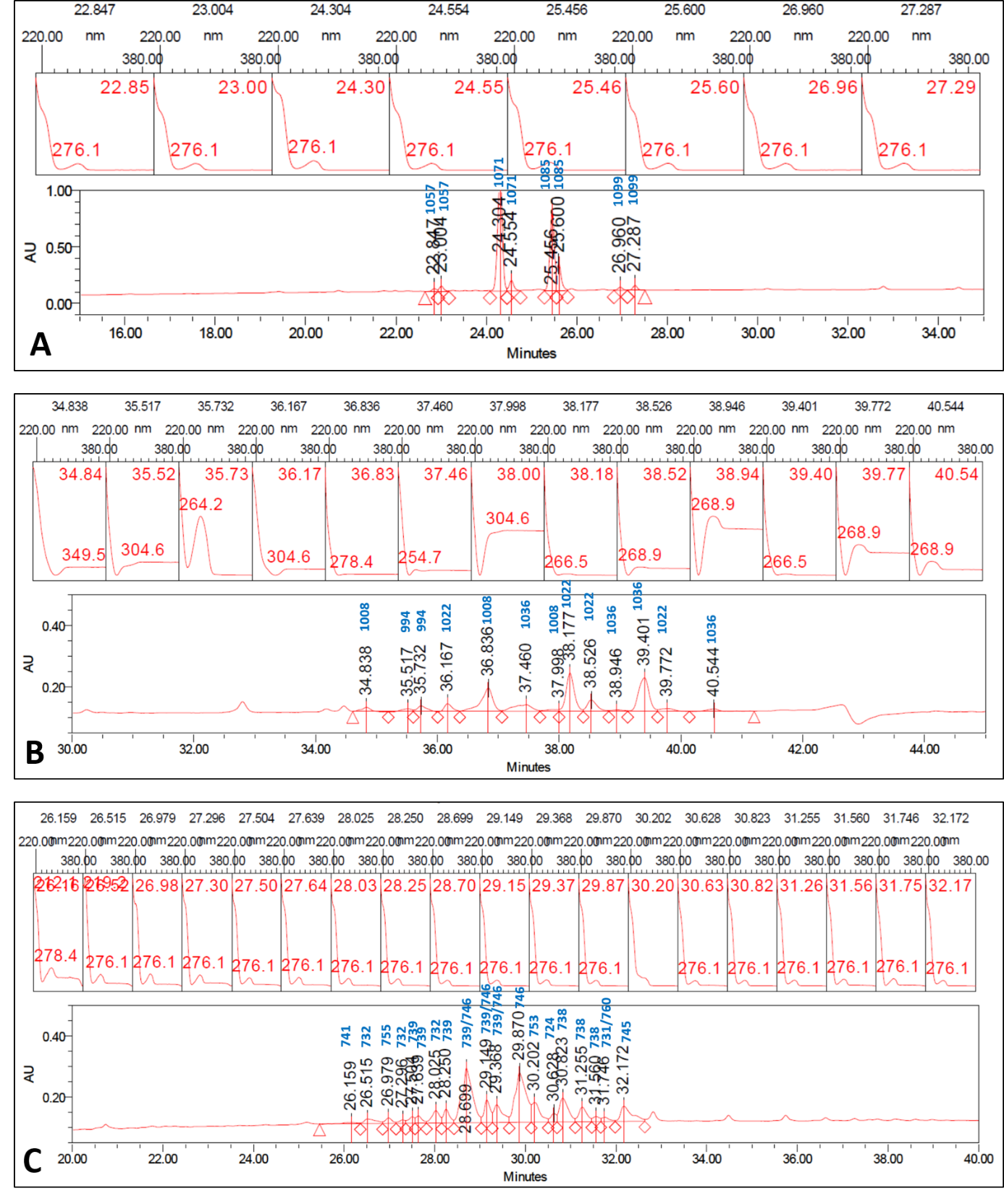
Figure S1: RP-HPLC-PDA-MS analysis of lipopeptide solutions (prepared at 1 g.L^-1^ in methanol): (A) mycosubtilins, (B) surfactins and (C) plipastatins.** For each figure the chromatogram displayed the UV-signal intensity (AU) at 220 nm and for each UV-peak, the retention time (min) and the molecular mass (blue color) of the eluted lipopeptide isoform.
